# Harnessing Tissue-derived Extracellular Vesicles for Osteoarthritis Theranostics

**DOI:** 10.7150/thno.62708

**Published:** 2022-01-01

**Authors:** Bohan Yin, Junguo Ni, Claire E. Witherel, Mo Yang, Jason A. Burdick, Chunyi Wen, Siu Hong Dexter Wong

**Affiliations:** 1 Department of Biomedical Engineering, the Hong Kong Polytechnic University, Hong Kong, 999077, China.; 2 Department of Bioengineering, University of Pennsylvania, PA 16802, USA.; 3 Research Institute of Smart Ageing, the Hong Kong Polytechnic University, Hong Kong, 999077, China.

**Keywords:** Osteoarthritis, Extracellular vesicles, Controlled-release, Biomaterials, Biosensors

## Abstract

Osteoarthritis (OA) is a prevalent chronic whole-joint disease characterized by low-grade systemic inflammation, degeneration of joint-related tissues such as articular cartilage, and alteration of bone structures that can eventually lead to disability. Emerging evidence has indicated that synovium or articular cartilage-secreted extracellular vesicles (EVs) contribute to OA pathogenesis and physiology, including transporting and enhancing the production of inflammatory mediators and cartilage degrading proteinases. Bioactive components of EVs are known to play a role in OA include microRNA, long non-coding RNA, and proteins. Thus, OA tissues-derived EVs can be used in combination with advanced nanomaterial-based biosensors for the diagnostic assessment of OA progression. Alternatively, mesenchymal stem cell- or platelet-rich plasma-derived EVs (MSC-EVs or PRP-EVs) have high therapeutic value for treating OA, such as suppressing the inflammatory immune microenvironment, which is often enriched by pro-inflammatory immune cells and cytokines that reduce chondrocytes apoptosis. Moreover, those EVs can be modified or incorporated into biomaterials for enhanced targeting and prolonged retention to treat OA effectively. In this review, we explore recently reported OA-related pathological biomarkers from OA joint tissue-derived EVs and discuss the possibility of current biosensors for detecting EVs and EV-related OA biomarkers. We summarize the applications of MSC-EVs and PRP-EVs and discuss their limitations for cartilage regeneration and alleviating OA symptoms. Additionally, we identify advanced therapeutic strategies, including engineered EVs and applying biomaterials to increase the efficacy of EV-based OA therapies. Finally, we provide our perspective on the future of EV-related diagnosis and therapeutic potential for OA treatment.

## Introduction

Osteoarthritis (OA) is a highly aging-related disease that involves entire joint disorder and is typically associated with irregular chronic pain, which seriously affects the life quality of patients 1. To some degree, almost all joint tissue abnormalities are involved in OA development. Articular cartilage as a pivotal part of joints plays an important role in OA progress 2. Cartilage is a resilient, elastic, and hydrated tissue that supports and cushions the ends of long bones at joints and does not contain vascular and neural networks 3. The primary cells found in cartilage are chondrocytes that produce extracellular matrix (ECM), including proteoglycans and collagens (Col). Cartilage can be divided into three major groups: fibrous cartilage, hyaline cartilage, and elastic cartilage in ascending order of ECM amount 4. Hyaline cartilage is the most widespread cartilage type found in different organs such as synovial joints, ribs, and trachea rings to tolerate bone loading and lubricate joint movement 5. Type II collagen (Col II) accounts for 90-95% of total collagen molecules and forms filamentous structures with collagen IX (Col IX) to resist tensile, and shear stresses in hyaline cartilage. Hence, the maintenance of ECM integrity is crucial for the regular function of cartilage. Damage, degeneration, or distortion of ECM elements and composition are the main features of cartilage diseases 6. Since chondrocytes are physically confined in lacunae and rely on diffusion to obtain nutrients due to the lack of blood supply 7, the matrix renewal process in cartilage is slow when compared to bone, and the damaged cartilage is easily susceptible to chronic diseases.

**Figure 1:**
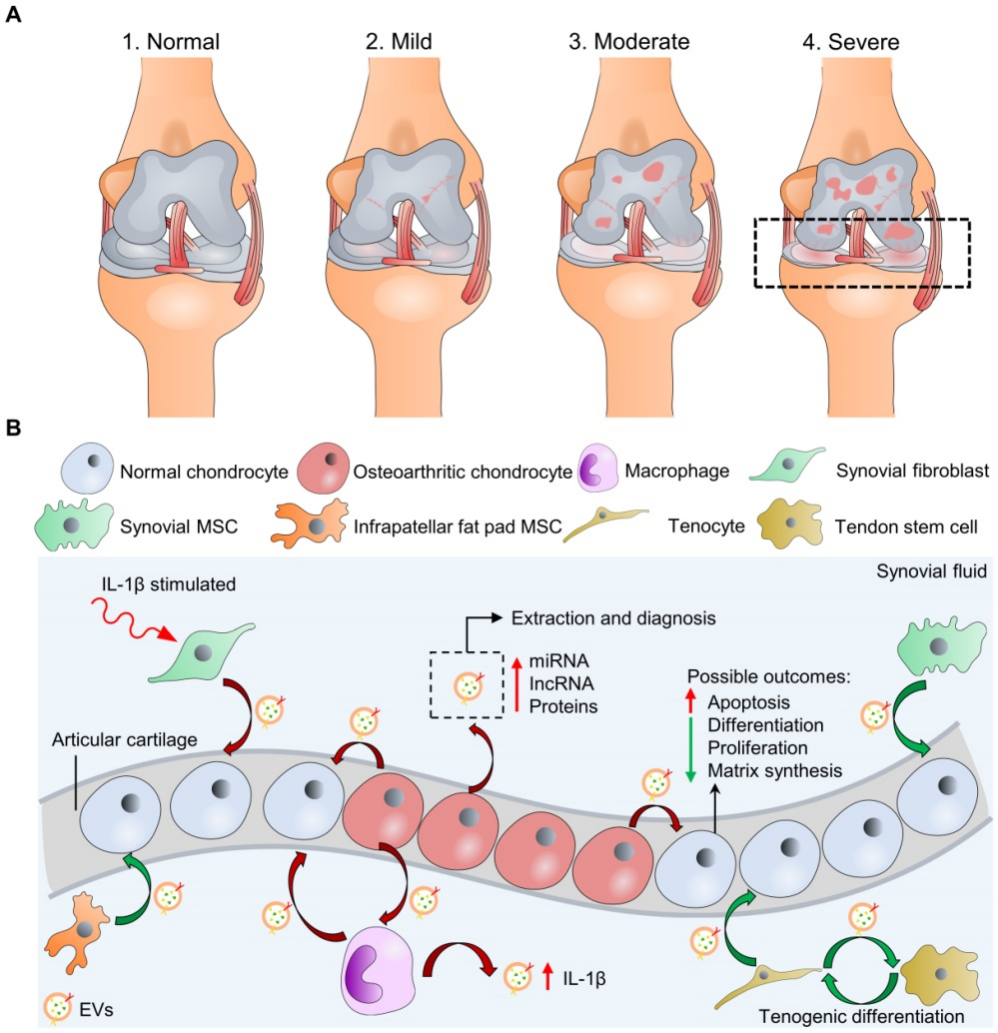
**Macroscopic and microscopic illustration of osteoarthritis (OA).** (A) Knee OA at four different stages can be evaluated by K-L scores, including “normal” at stage 1. Only cartilage degradation is shown for illustration simplicity instead of the whole-joint damages. (B) Microscopic exploration of normal and osteoarthritic chondrocytes interacting with other cell types through cell-cell communication (EV secretion) at stage 4 with possible biological outcomes. The synovial fluid-derived EVs can be extracted for OA diagnosis. Green/red curve arrows indicate cells secreting EVs with bioactive molecules that potentially are chondro-protective/chondro-destructive. Osteoarthritic chondrocytes may also secret EVs to stimulate inflammasome activation of cells in synovial space, including macrophages.

The degeneration of articular cartilage is referred to as one of the hallmarks of OA 2, 8. Traumatic injuries, obesity, and congenital abnormalities are the clinically relevant causes of pathological conditions that undermine cartilage load-bearing capacity and lead to chronic diseases like OA 9. Pathological conditions such as inflammation often perturb the microenvironment of cartilage ECM, resulting in dysfunction and apoptosis of chondrocytes, which further aggravates OA 10. According to the Kellgren-Lawrence classification system (K-L score) by radiography, stages of knee osteoarthritis can range from (1) normal, (2) mild, (3) moderate, and (4) severe stages (**Figure 1A**) 11. K-L scores 1-2 are defined as early-stage OA, and K-L scores 3-4 are late-stage OA 12. During OA, the components of the joint tissues, including bone, joint capsule, synovial tissue, tendons, ligaments, and cartilage, fail in various ways, leading to joint instability 13. One of the significant phenomena is that the cartilage surface progressively erodes, and joint inflammation becomes severe along with the increased stage level and is accompanied by a systemic low-grade chronic inflammation. However, the self-healing process of damaged articular cartilages is slow and limited, as mentioned previously. Hence, in the treatment of OA, articular cartilage has mainly been the focus of research. Current treatment options, including surgical (e.g., total knee replacement, TKP) and non-surgical (e.g., pharmaceutical treatment, viscosuppements) therapies, are associated with side effects and low-efficacies 14-18. To overcome these limitations, cell-based therapies have been suggested to replace or stimulate endogenous regeneration of damaged cartilage tissue. Stem cells, including mesenchymal stem cells (MSCs) demonstrating multipotency, and embryonic stem cells (ESCs), and induced pluripotent stem cells (iPSCs) demonstrating pluripotency, can undergo differentiation into somatic cells of the organ that are critical to restoring and repairing injured tissues 19. However, cell-based therapies may require operational surgery and high costs to maintain large cell numbers before the final delivery to patients. Moreover, previous studies have reported that engrafted stem cells in a diseased joint environment with inflammatory cytokines can intensify the inflammatory response and escalate disease progression 20-22. Even though MSCs from the same tissue of origin have been characterized with strong immunomodulation and inflammatory suppression ability, they have demonstrated prodigious batch-to-batch and donor-to-donor variation that can influence MSC availability and function 23, 24. The existence of MSC heterogeneity is potentially the reason for this variation that different subpopulations can show distinct expression profiles and functional properties from the same sample source 25-27. Thus far, improperly purified MSCs may induce adverse immune effects upon injection to the OA site.

As an alternative, extracellular vesicles (EV) or exosomes that are produced by stem cells and contain potent cytokines, growth factors, and miRs may be powerful in mediating inflammation and enhancing progenitor cell proliferation 28. The benefits of MSC-derived EVs in treating cardiovascular, respiratory, renal, and hepatic diseases 29-31 and cartilage regeneration are well-established 32. EVs exhibit an increased capacity to escape degradation or clearance by the immune system 33, and MSC-derived EVs (MSC-EVs) have been shown to play substantial therapeutic roles in regulating intracellular pathways in different diseases, including inflammatory bowel disease 34, 35, neurodegenerative diseases 36, 37, and respiratory tract diseases 38, 39 or pneumonia infections related to COVID-19 40, mainly due to their immunomodulatory effects, including suppression of inflammation.

Apart from building up effective treatment strategies, early diagnosis of OA is of pivotal importance to limit further progression of cartilage damage 41. Therefore, investigating biomarkers of early-stage OA helps prevent the disease progression and potentially probes the initial molecular mechanisms that lead to OA initiation. In this review, we aim to introduce (1) the biological characteristics of body tissue-derived versus OA tissue-derived EVs, (2) synovial fluid-derived EVs and their OA biomarkers that can be detected by currently available biosensors, (3) therapeutic values of MSC-derived EVs for treating OA, and (4) highlight the cutting-edge technologies and discuss the current limitations of EV-based and biomaterial-based platforms toward optimizing cartilage/OA therapy.

## The biological and pathophysiological characteristics of EVs

Extracellular vesicles (EVs) are membrane vesicles with diameters of 30-5000 nm secreted from various cells that communicate with each other *via* paracrine signallings 42. The term EVs is often used as an umbrella term but can be further broken down into different terms associated with specific sizes. Apoptotic bodies are considered the largest EVs with diameters from 1000-5000 nm, extracellular microvesicles range in size from 100-1000 nm, while exosomes (also known as small EVs) are typically defined by diameters of 30-150 nm vesicles 43. EVs can be generally separated by ultracentrifuge from body fluids/whole blood or the culture medium during cell culture. To obtain small EVs from the other EV subpopulations (e.g., apoptotic bodies), size-based separation methods such as filtration, flow field-flow fractionation, affinity-based techniques, and size-exclusion chromatography (SEC) have been adopted for the separation 44. The studies featured in this review primarily focus on exosomes or small EVs, and we use EV(s) to describe them, according to minimal information for studies of extracellular vesicles (MISEV) recommended by the International Society for Extracellular Vesicles (ISEV) 45. The paracrine signalling requires transferring donor (EV-secreting cells) cargo to recipient cells by exocytosis and endocytosis, respectively 42. Specifically, EVs contain and protect useful biological information, including long non-coding RNAs (lncRNAs), messenger RNAs (mRNAs), regulatory microRNAs (miR), lipids, and proteins. Such information transportation facilitates non-contact intercellular communication, thereby regulating the behaviours of distant cells. Therefore, EVs are clinically significant for biological signal transmission and as promising natural nanocarriers for clinical application. The biogenesis and isolation procedures of EVs have been comprehensively reviewed by others and will not be discussed in detail in this review 42, 46-48.

### Overview of EV characteristics from cartilage and chondrocytes

EV-related materials in the pericellular cartilage matrix and growth plate cartilage have long been described in the previous literature 49, 50. Generally, EVs bear surface markers CD9, CD63, CD81, LC3, tumour susceptibility gene 101 (TSG101), flotillin-1, and Alix, although a recent study reveals that CD63 is a more specific EV surface marker compared to others and non-CD63 bearing vesicles can be ectosomes 51. The reported size and surface markers of human articular chondrocyte-derived EVs are similar to that of other cell-derived EVs. Although some reports highlighted that heterotrimeric G protein, HSP70 and 90, and members of the tetraspanin family such as CD9, CD63 and CD81 were not detected in the proteome of articular cartilage-derived EVs 52, 53, emerging research confirmed that CD9, CD63, and TSG101 proteins were expressed on chondrocyte-derived EVs 54-57. Meanwhile, the content of EVs varies along with different types of tissues. In 1969, Anderson identified matrix vesicles (size from ~30 nm to 1 µm) containing hydroxyapatite and/or fluorapatite at all levels in the epiphyseal plate of calcified cartilage 58. The matrix vesicles were later discovered to enrich in phosphatase that hydrolyzed a variety of nucleotide triphosphates, diphosphates, monophosphates, and other phosphate-containing substrates and metabolites to facilitate the precipitation of hydroxyapatite for calcification 59. These findings demonstrated that cartilage calcification is associated with the deposition of apatite-like material, including the matrix vesicles, to bind calcium for endochondral bone development. Also, the results imply that EVs may possess a high tissue-penetration ability to diffuse deep into cartilage for delivery. Indeed, more than 1,700 proteins and mRNAs for factor XIIIA, type II transglutaminase, collagen II, aggrecan, ANKH, and GAPDH were identified in articular cartilage-derived EVs 52, 60. The Articular cartilage-derived EVs have been shown to concentrate those enzymes (e.g., coagulation factor XIIIA and metalloproteinase), ions, and substrates necessary for mineral formation, implying that they can be considered as physiologic structures in articular cartilage 61. Specifically, there are quantitative changes of matrix proteoglycans and TGF-β signalling pathway-related proteins in OA 62. Nevertheless, these changes might contribute to the reactivation of ossification centres and matrix mineralization of articular cartilage, one of the hallmarks of OA 63.

### Diagnostic value of EVs in OA

Recent research has illustrated the involvement of EVs in the pathological and physiological processes of OA 64. Pathological EVs play a crucial role in inflammation and chronic pain diseases and have emerged as a potential marker in OA 65. EV cargoes from OA pathological conditions may show distinct genomic and proteomic profiles for distinguishing pathological EVs from physiological EVs that help identify OA at an early stage. Moreover, previous studies have focused on the diagnostic significant and biological outcomes of endogenous EVs during OA (**Figure 1B**). For instance, evidence showed that OA chondrocytes (from OA patients undergoing total knee replacement, TKR) actively released EVs with enriched miR-372 lncRNA and a low level of a lncRNA, HULC (highly upregulated in liver cancer), while EVs from normal articular chondrocytes showed a reverse trend 66. Similarly, OA chondrocytes (from OA patients undergoing TKR) produced EVs with high content of miR449a-5p to inhibit autophagy (a function that eliminates unwanted materials and suppresses inflammasome activation) and promote mature IL-1β production of macrophages 67. This process was shown to aggravate synovitis in the destabilization of the medial meniscus (DMM) OA model in knee joints of 8-week-old male mice. Other cell types of joint tissues, including synovial fibroblasts, synovial MSCs, infrapatellar fat pad MSCs, or tenocyte/tendon stem cells, may also interact with normal/OA chondrocytes *via* releasing EVs in the synovial space. Ni et al. and Withrow et al. have summarized these interactions that can be referenced for probing OA pathogenesis 62, 68.

Several types of EV readouts, including the size, amount, and biological contents of EVs from the diseased sites, can be the representative biomarkers. Mustonen and colleagues recently showed that synovial fluid (SF) from the human knee joint with rheumatoid arthritis (RA) has a significantly higher proportion of hyaluronan (HA)-positive EVs at size range 101-200 nm but a much lower proportion of HA-positive EVs at size range >501 nm than those in OA and control groups 69. This finding provides a valuable reference for the polydispersity of EVs size and surface bioactive moieties from different disease sites. Similarly, Xu et al. determined that SF from early-stage and late-stage OA in patients contained a higher amount of EVs than those in control groups 70. Specifically, the expression level of lncRNA, PCGEM1, an OA-related marker (a sponge for miR-770 for stimulating the proliferation of OA synoviocytes) in EVs, was remarkedly higher in the late-stage OA group than those in the early-stage OA group, suggesting that different stages of OA can be distinguished by analyzing EV contents from SF.

Non-coding RNA is an important biologically active molecule in EVs, including miR, lncRNA, and circular non-coding RNA (circRNA) 14. Increasing evidence proves that they play critical roles in regulating the occurrence and development of diseases 15, 16. Proteins in EVs from the synovial fluid are another critical biomarker responsible for cell-to-cell communication in the OA microenvironment 71, 72. These non-coding RNA and proteins are either upregulated or downregulated during the occurrence of OA or RA that deteriorates the normal functions of articular chondrocytes. Emerging evidence has suggested that these bioactive signals are transmitted *via* EVs. For instance, Liu and colleagues have demonstrated that synovial fibroblast secreted EVs containing miR-126-3p are responsible for suppressing apoptotic cell death, inflammation, and osteophyte formation in chondrocytes, and this miR expression was significantly reduced in OA patients 73. Similarly, a lncRNA, the upregulation of PVT1 expression was shown in isolated EVs from whole blood of OA patients, and PVT1 regulated OA progression through the HMGB1/Tlr4/NF-κB signalling pathway 74. Another study revealed that protein profiles of SF-derived EVs in RA, axial spondyloarthritis, gout, and OA patients were different 75. For instance, haemoglobin and actin-related protein 2/3 complex subunit 3 in EVs were more abundant in the OA group compared to the other three groups. We have tabulated OA-related EV-derived biomarkers, including microRNA, lncRNA, and proteins, from the recent studies as useful references for OA detection (**Table 1**). Therefore, it is highly desirable to develop biosensors, especially with current advances in nanotechnology and biomaterials, for detecting OA-related EVs and the EV contents to probe the progression of OA 76.

Despite the biochemical contents of EVs, biophysical properties of OA-related EVs have been rarely explored. A recent report revealed the mechanical difference between non-malignant and malignant cell lines EVs by employing quantitative nanomechanical mapping atomic force microscopy (QNM AFM) 77. The authors isolated EVs from human urothelial HCV-29 cells (non-malignant cells), human urothelial FL3 cells (malignant cells), and non-metastatic parental cell line T24 (malignant cells). Intriguingly, QNM AFM results showed that EVs derived from a non-malignant cell line (HCV-29: ~1527 MPa) were stiffer than those from malignant cell lines (FL3: ~280 MPa and T24: ~95 MPa). Similarly, malignant cell-derived EVs exhibited a higher adhesion force to the AFM tip than that of the non-malignant cell-derived EVs, suggesting an increased interaction between the tip and EV surface constituent. Consistently, the reduced stiffness in malignant cells (HCV-29 and T24)-derived EVs correlated with the reduced cell stiffness by order of magnitude that might contribute to the ability of EVs to transport across biological membranes 78. Based on these findings, we may expect that EVs derived from osteoarthritic chondrocytes can be softer than that of normal chondrocytes for an increased tissue-infiltration property, leading to OA progression deteriorating. This postulation needs further justification.

### Biosensors to detect EV-based biomarkers to monitor OA progression

Plain radiography is traditionally the gold standard for morphological assessment of OA knee with K-L score analysis of the images 12. However, this method may only detect the cartilage change with >10% cartilage lost and cannot be able to visualize other soft tissues, including meniscus and ligaments in the joint. Magnetic resonance imaging (MRI) is one of the reliable methods to detect the damaged cartilage in OA anatomically with ~70% sensitivity and 90% specificity, compared to reference diagnosis by arthroscopy (invasive approach to observe joints) 83. However, MRI techniques require expensive equipment, lengthy processing time and are not suitable for those patients implanted with metallic devices such as pacemakers. Also, the diagnostic standard of MRI-based OA needs further clinical validation. Thus, serological tests may provide an alternative option to detect biochemical changes in serum/SF of patients with OA/RA at the early stage 84, 85. For molecular biomarkers in RA, anti-cyclic citrullinated peptide (CCP, plays a critical role in initiating inflammatory responses in autoimmune diseases, such as RA) is a biochemical marker for detecting early-stage RA and the reported sensitivity and specificity by serological tests were ~60% and ~90%, respectively 86. A recent study developed an isotopic dilution analysis mass spectrometric method to analyze the concentration of citrullinated peptide (CP), anti-CCP, and 4-hydroxyproline (Hyp, a marker of bone turnover and resorption) by biochemical assay to discriminate the type of arthritis at the early stage (within five months of the onset of symptoms of inflammatory arthritis) and advanced arthritis stage in plasma/serum/synovial fluid of patients 87. The key findings of this study show that the stage and the type of arthritis (OA or RA) can be identified by measuring the amount of CP and Hyp in serum and synovial fluid and match expression patterns to reported data from this study. Nevertheless, further research is required to investigate whether these markers can be found in EVs from OA sites.

**Figure 2:**
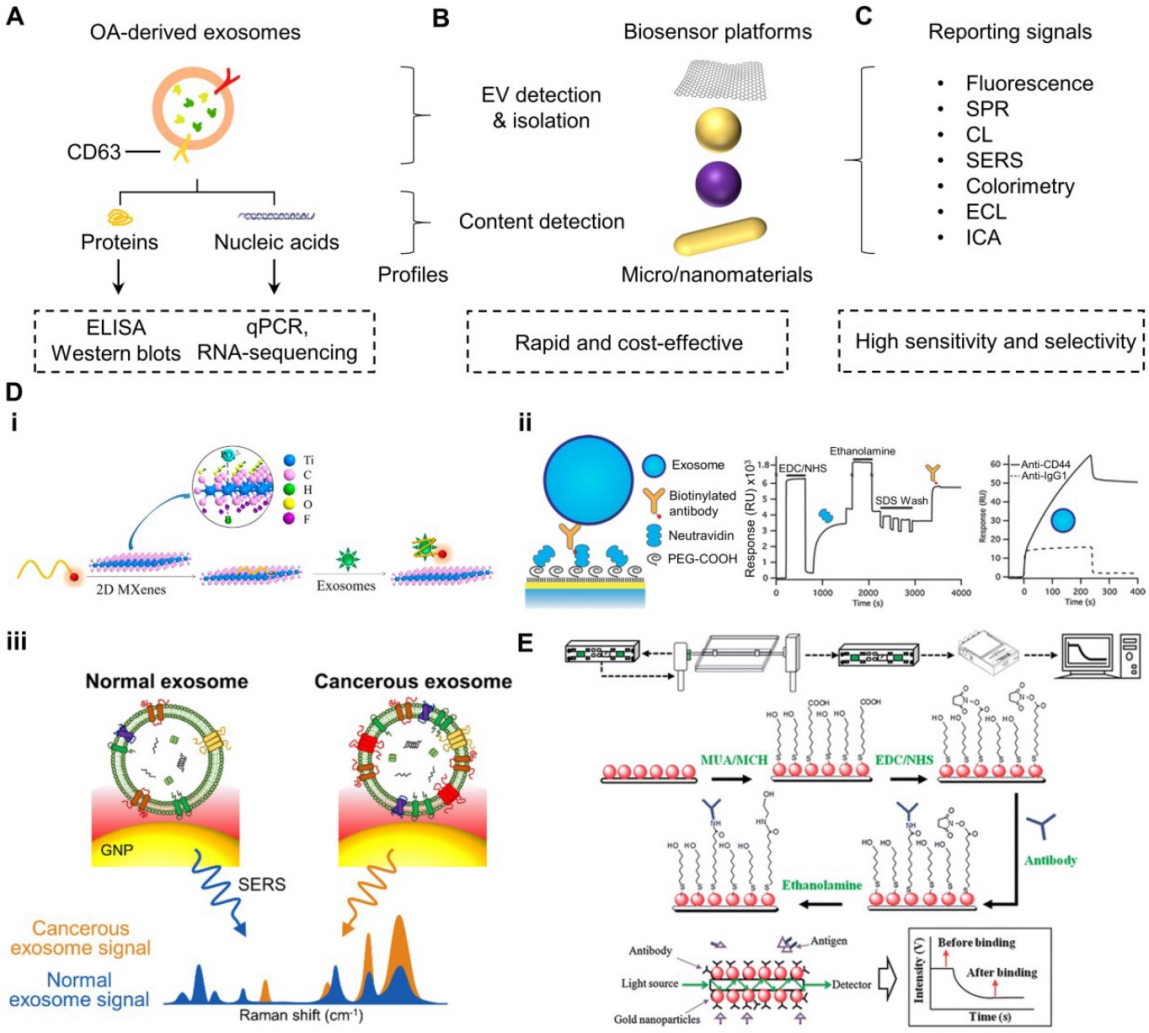
**Overview of the process and principles of biosensors to detect EVs and OA biomarkers in EVs.** (A) OA site-derived EV proteins and nucleic acids can be conventionally detected by ELISA-based and qPCR-based methods, respectively. (B) Recent advances in nanotechnology develop many rapid and cost-effective biosensors for detecting EVs and EV contents through (C) various techniques. (D) EVs can be probed by (i) fluorescence-based system on CD63-targeting Cy3-conjugated aptamer, which is initially quenched by MXene nanosheets and recovers the fluorescent signal upon binding to EVs in the solution 79; (ii) SPR-based system with EV surface protein-specific antibodies to capture EVs that cause the evanescent surface plasmon wave at the sensor surface to generate differential optical signals for distinguishing normal or diseased EVs 80; and (iii) SERS-based system to amplify the Raman profile of specific surface protein of EVs for distinguishing normal or diseased EVs 81. (E) EV contents can also be detected by biosensors, such as the use of an SPR-based system consisting of specific antibodies immobilized on gold nanoparticles/optic fiber sensor to detect OA-related markers (such as TNF-α) from human knee SF 82. The figures are reprinted and re-arranged with permission from Ref. 79-82. Copyright American chemical society. (2018), Royal Society of Chemistry (2013), and SpringerLink. (2015).

A number of analytical assays have been employed to detect EV sizes by nanoparticle tracking analysis (NTA) and EV contents, including the mentioned biomarkers by quantitative reverse transcription-polymerase chain reaction (qRT-PCR) and RNA-sequencing (RNA-seq) for nucleic acids 88, 89, flow cytometry and magnetic bead-based isolation for EV isolation 90, 91, enzyme-linked immunosorbent assay (ELISA) and western blotting (WB) for proteins (**Figure 2A**) 92, 93. Although these methods have been robust and highly reliable, limitations such as time-consuming operations and complicated procedures are noted 94. Rapid and sensitive biosensors with simple handling steps are alternative and promising choices to probe OA biomarkers in EVs isolated from OA sites, such as SF.

Numerous studies applied nanomaterials typically conjugated with anti-CD63 antibody, CD63 aptamer, or EV-related surface marker capturing molecules to bind EVs and switch on particular physical or chemical signals, including fluorescence 79, 95, 96, surface-enhanced Raman scattering (SERS) 97-99, surface plasmon resonance (SPR) 100, 101, colorimetry 102, 103, immunochromatographic assay (ICA) 104, chemiluminescence (CL) 105, 106, and electrochemiluminescence (ECL, **Figure 2B-D**). For instance, Zhan et al. developed a self-standard ratiometric fluorescence resonance energy transfer (FRET) nanoprobe, consisting of Cy3-CD63 aptamer adsorbed onto 2D MXene nanosheets (fluorescent quencher) *via* hydrogen bonds and metal chelate interactions for quantifying the EVs in the solution (**Figure 2Di**) 79. The detecting mechanism was based on the initial “OFF” Cy3-CD63 aptamer fluorescent signal quenched by MXene, but the aptamer was specifically bound to CD63 protein of EV surfaces, thereby loosening the attachment to MXene and recovering fluorescent signal of Cy3-CD63 as “ON” state. The detection time only required 1 h. The reported limit of detection (LOD) for EVs by this platform was 1.4 x 10^3^ particles mL^-1^, which was 1000x lower than that of ELISA. This method offers a rapid and ultrasensitive approach to detect EVs. On the other hand, Shin and colleagues reported a SERS-based platform comprised of aggregated and positive charge gold nanoparticles (AuNPs) coated on a glass substrate for capturing the negative charge surface of EVs (**Figure 2Diii**) 81. Their results showed that SERS fingerprints of the proteins on EV surfaces were intensified by the localized SPR of the substrate. Importantly, their findings demonstrated that nonsmall cell lung cancer-derived EVs dominantly expressed epidermal growth factor receptors on their surface but not on regular cell-derived EVs, resulting in unique Raman scattering profiles for cancer diagnosis. This platform can potentially be useful to distinguish OA chondro-derived EVs from the normal EVs, although limited reports suggest any surface proteomic difference between OA chondro-derived EVs and normal chondro-derived EVs. To develop an SPR biosensor specific to surface biomarkers of EVs, Grasso et al. constructed a real-time and label-free EV monitoring platform to identify the molecular profile of EVs from cultured cell lines or isolated from human biofluids (**Figure 2Dii**) 80. This platform consisted of gold-coated sensor surfaces conjugated with antibodies specific to CD44, CD63, CD24, CD9, epithelial cell adhesion molecule (EpCAM), or human epidermal growth factor receptor 2 (HER2). The detection mechanism was based on the evanescent surface plasmon wave at the contacting dielectric region of the gold sensor surface. Thus, this biosensor quantified changes in the number of cancer cell-specific EVs from human blood at the sensor surface upon binding to the antibodies within 1 h. These studies demonstrate the importance of developing advanced biosensors to replace conventional methods that are time-consuming and multiple handling steps. Several excellent reviews have also comprehensively discussed the applications, strengths, and drawbacks of the nanomaterial-based biosensors for detecting EVs 98, 107, 108. After those nanoplatforms identifying and isolating EVs, EV-derived biomarkers can be profiled by conventional methods.

Similarly, recent reviews have discussed various biosensors for detecting OA and RA biomarkers based on the mechanisms above for signal amplification 76, 109, 110. For example, Huang and colleagues reported a label-free and real-time fiber-optic particle plasmon resonance sensing system, of which the fiber outer surface is coated with antibody-bearing AuNPs (**Figure 2E**) 82. The optic fiber restricted light to pass as a multiple total internal reflection scheme that permitted a high contact chance between the light and AuNPs to enhance the SPR signal-to-noise ratio. The LODs achieved by this platform for TNF-α and metalloproteinase-3 (MMP-3) in human SF from knee joints of OA patients (12 patients) were 8.22 pg mL^-1^ (0.48 pM) and 34.3 pg mL^-1^ (1.56 pM), respectively. This platform was more sensitive and more rapid (< 10 min) than that of the conventional ELISA (cut-off LOD at ~100 pg mL^-1^ with handling time ~6 h). These findings demonstrate the possibility of probing OA biomarkers by rapid and sensitive biosensors. Point-of-care, non-invasive, and real-time biosensors can be highly attractive for clinical applications towards screening OA. However, very limited literature reports the usage of biosensors to detect the content of EVs from OA joints. Moreover, biosensors that can simultaneously probe both EV membranes and EV-associated contents in fluid samples in OA patients have been rare. We believe that biosensors with this detection ability are novel, attractive, and cost-effective without the need for EV isolation to understand the stage of OA, as OA-related markers can be concentrated in EVs.

## The therapeutic value of non-bone marrow MSC-derived EVs for OA

MSC-EVs exhibit great therapeutic potential for treating OA, as previous literature shows that the paracrine factors of MSCs provoke chondrocyte proliferation 116. The biology, preparation, characterization, and applications of MSC-EVs have been extensively discussed in previous reviews 14, 117-119. We have enlisted several recent reports utilizing bone marrow MSC-EVs to treat OA in different animal models (**Table 2**). However, the isolation of bone marrow MSCs from other tissues requires invasive procedures that increase pain and cost for the patients. Therefore, alternative sources for isolating EVs from other tissues, including synovial MSCs, platelet-rich plasma, infrapatellar fat pad, and umbilical cord-derived MSCs, have been emerging and showing promising tissue engineering results and treating/inhibiting OA symptoms. This section highlights and explores the recent findings of EVs derived from non-bone marrow MSC sources as potential options for treating knee OA and cartilage injuries.

### Platelet-rich plasma-derived EVs

Platelet-rich plasma (PRP) is an autologous derivative of whole blood 120. The blood can be centrifuged and separated into the following components: plasma, platelets, and leukocytes (the “buffer coat”), and erythrocytes from top to bottom layers. The preparation of PRP is generally based on its leukocyte and fibrin content ratio with four categories: (1) leukocyte-rich PRP (L-PRP); (2) leukocyte reduced PRP (P-PRP); (3) leukocyte platelet-rich fibrin; and (4) pure platelet-rich fibrin 121. PRP is demonstrated to play critical roles in bone and soft tissue healing processes 122. Numerous studies have reported regenerative and anti-inflammatory effects of PRP administration to the sites of advanced-stage diseases such as OA 123-125. Mechanistic studies have revealed that activated platelets secret a high amount of growth factors (GFs) and cytokines to promote cell proliferation and inhibit the apoptosis of chondrocytes 126, 127. This secretion can be mediated by delivering EVs that interact with chondrocytes for fusion and subsequent release of bioactive contents 128. However, it is shown that leukocytes-containing PRP (e.g., L-PRP) can concentrate pro-inflammatory cytokines, thereby showing less effective OA treatment *in vivo* than that of P-PRP 126. On the other hand, several leukocyte subsets, including M2 macrophages showing anti-inflammatory may initiate tissue repair and suppress fibrosis 129. Thus, the inclusion of leukocytes in PRP formulations is debatable. Leukocytes can be removed from PRP to avoid complications.

A recent study demonstrated that activated PRP upregulated the expression of platelet-derived growth factor-AB (PDGF-AB), transforming growth factor-β (TGF-β), and vascular endothelial growth factor (VEGF). These factors were secreted in PRP-EVs to promote cell proliferation (with reduced apoptosis) and cartilaginous matrix secretion *via* suppressing the Wnt/β-catenin signal pathway in interleukin-1β (IL-1β)-stimulated chondrocytes, which were harvested from the terminal of tibia and femur of 4-week-old New Zealand white rabbits 130. Notably, this study reported intriguing findings that the accumulation of β-catenin and Wnt5a increased IL-1β-induced osteoarthritic chondrocytes but could be reversed by the presence of PRP-derived EVs (PRP-EVs) or activated PRP. Importantly, the PRP-EVs also reduced the expression level of tumour necrosis factor-α (TNF-α), a pro-inflammatory mediator of OA. The authors further showed that intra-articularly injected PRP-EVs induced more cartilage repair and OA inhibition than activated PRPs alone in a rat OA model (6-7 weeks post-surgery), which was created by cleavage of the medial collateral ligament and the anterior cruciate ligament with the excised medial meniscus in the left knee of the rabbits. This study demonstrates a novel strategy to utilize PRP as an EV source to treat damaged cartilage.

### Infrapatellar fat pad MSC-derived EVs

Human infrapatellar fat pad (IPFP)-derived MSCs can generally be obtained by a single arthroscopy during knee arthroplasties 131. Previous literature has reported that the three-dimensional co-culture of IPFP MSCs with articular chondrocytes (ACs) in the same cell number ratio promotes chondrogenic outcomes and prevents the inflammatory status of ACs and hypertrophic differentiation of MSCs 132. For example, their results showed that the concentration of the secreted 1L-1β and MMP-13 declined during the co-culture, especially with the presence of chitosan/hyaluronic acid nanoparticles (NPs). However, a previous study illustrated that IPFP-derived MSCs or synovial fluid-derived MSCs expressed human leukocyte antigen-DR (HLA-DR) under interferon-gamma (IFN-γ) stimulation, which was particularly enriched in the OA microenvironment. This HLA-DR interacts with T-cells *via* MHC class II molecules that potentially trigger an alloresponse, rejecting foreign transplanted cells 131, 133. Large-scale allogeneic therapies require a large cell number limited to IPFP-MSCs isolation from the donor and hence hampers the practical application of IPFP-MSCs for OA therapy.

Recently, Wu et al. reported that EVs derived from IPFP (IPFP-EVs) contained abundant miR-100-5p, as evident by RNA-sequencing (RNA-seq) 134. Their findings showed that the injection of IPFP-derived EVs into the articular space of the DMM mouse model alleviated OA severity with low OARSI (Osteoarthritis Research Society International) grade compared to the PBS (phosphate buffer saline) control group, inhibited cell apoptosis, enhanced matrix synthesis (e.g., Col II), and reduced the expression of catabolic factors (e.g., MMP13) *in vitro* and *in vivo*. Moreover, their mechanistic study showed that miR-100-5p from IPFP-EVs was able to bind to the 3'-untranslated region (3'UTR) of mTOR that inhibited the autophagy activity signalling pathway, such as the expression of ADAMTS5 and MMP13 that are responsible for OA progression. Therefore, IPFP-EVs may provide an alternative therapeutic source for treating OA.

### Synovial MSC-derived EVs

Synovial MSCs (SMSCs) have a remarkable proliferative and chondrogenic potential for cartilage repair 135. SMSCs can be simply isolated from the synovium of human knee joints by fluorescence-activated cell sorting (FACS). It has been shown that the injured joint and OA knee induces the mobilization of MSCs into the synovial space 136. The possible source of SMSCs is the synovium, although no direct evidence shows this process. The therapeutic value of SMSC-derived EVs (SMSC-EVs) has been frequently explored 137. For instance, Lian and colleagues have demonstrated SMSC-EVs containing miR-31 that play inhibitory roles in the regulation of lysine demethylase 2A (KDM2A), which associates with the demethylation of histone H3 at lysine 4 (H3K4) at the secreted frizzled-related protein 2 (SFRP2) 138. SFRP2 is shown to inhibit osteogenesis and induce the occurrence of OA 139. Critically, their findings denoted that KDM2A suppressed proliferation and migration of articular chondrocytes through binding to the transcription factor E2F transcription factor 1 (E2F1), which promoted the expression of pituitary tumour transforming gene 1. Therefore, the delivery of SMSC-EVs into the OA model reduced cartilage damage with downregulation of IL-1β, IL-6, and TNF-α expression and activated E2F1/PTTG1 axis to prevent the occurrence of knee OA. This report has shed light on SMSCs-EVs mediated signalling pathways for treating OA.

To further enhance chondrocyte ECM secretion *via* SMSC-EV delivery, Wang et al. overexpressed SMSCs with miR-155-5p and harvested their EVs (SMSCs-155-5p-EVs) as the miR-155-5p enriched cargoes 140. This study illustrated that SMSCs-155-5p-EVs exerted an inhibitory effect on Runx2 expression and an elevation effect on the expression of ColII (collagen II) and SOX9 in the stimulated chondrocytes and OA model of BALB/C mice. A similar study showed that the overexpressed miR-140-5p in SMSCs-EVs targeted ras-related protein (RalA) to promote SOX9 and aggrecan translation 135. These results imply that the delivery of EVs from the cells with overexpressing genes critical for miR biogenesis and processing can assist the activation of multiple signalling pathways to alleviate OA symptoms and promote cartilage repair 141. Nevertheless, the limited number of mobilized SMCSs present in the synovial fluid restricts the contribution to repairing diseased injuries by natural processes 136 and hence leads to a low yield of EVs that refrains their translation research.

### Umbilical cord tissue-derived EVs

Human bone marrow-derived MSCs (hBMMSCs) are the most commonly used MSCs for research and clinical purposes. However, there are some limitations of hBMMSCs. For example, the relative number of hBMMSCs in the marrow and their differentiation potential decreases significantly with the age of donors 142. Also, the isolation procedure is painful and invasive that may cause complications and morbidity to donors 143. Recently, umbilical cord tissue-derived mesenchymal stromal cells (UCMSCs) have been an emerging MSC source that overcomes these limitations, as the harvesting procedure is not invasive or painful and does not involve donor site morbidity, according to the isolation instructions (enzyme digestion or explant culture method) from Wharton's jelly umbilical cords 144. Also, it is reported that the primary UCMSCs can be expanded ~300 times of the original cell number for more than seven passages without the loss of differentiation potential 145, thereby lowering the cost for yielding the same amount of EVs from hBMMSCs. Several studies also demonstrate the excellent potential of UCMSCs to differentiate into chondrogenic lineage for cartilage tissue engineering 146, 147. Based on the previous descriptions of the advantages of umbilical cord-derived EVs over adopting MSCs alone, recent research has demonstrated the application of UCMSC-derived EVs (UCMSC-EVs) for osteochondral regeneration and joint arthritis.

Yan et al. have shown that a rotary cell culture system creates microgravity for the 3D culture of UCMSCs by cell aggregation 148. Their findings illustrated that such 3D culture yielded more UCMSC-EVs than those in 2D culture. More importantly, the EVs contained a high level of lncRNA H19, a highly conserved sequence with ~2.3 kb in length to play an important role in the osteochondral activity, stem cell differentiation, and embryonic growth. In this study, the authors demonstrated that UCMSC-EVs promoted chondrocyte proliferation and matrix synthesis (e.g., collagen II and aggrecan) and inhibited apoptosis *in vitro* with IL-1β stimulation (osteoarthritic chondrocyte model). Also, they employed a rat cartilage defect model with a drill bit to assess the efficacy of UCMSC-EVs treating damaged cartilage tissues in vivo. Their results further showed that UCMSC-EVs treated model exhibited the highest score by International Cartilage Repair Society (ICRS) macroscopic assessment and the highest pain persistent level, compared to those in the PBS control group and H19 silencing UCMSC-EVs group. A similar study was reported by the same group employing a hollow-fiber bioreactor (cylindrical fibers) to simulate 3D culture for enhancing the production of UCMSC-EVs 149. Thus, UCMSC-EVs show great potential for cartilage defects other than conventional BMMSCs.

### Limitations

Although non-MSC sources can be considered as alternative options for harvesting regenerative EVs, several limitations arising from the cell source and methodology of EV isolation may restrict the application of these sources. For instance, the volume of autologous concentrates of PRP is limited, and the protocol of harvesting PRP lacks reproducibility as the separation methods may not be standardized 150. Other confounding factors include donor variability, storage conditions, or the use of external activators, including calcium and thrombin can limit the clinical use 151-153. Besides, it is reported that EV isolation methodology can influence the biological effects of PRP-EVs and their clinical translation. Specifically, lipoproteins usually accompany PRP-EV during the isolation process (e.g., ultracentrifugation as the traditional method) and hence decrease the PRP-EV purity as lipoproteins may impose pro-inflammatory effectors that cause undesirable effects 154. In fact, other challenges to harvesting tissue-derived EVs are those EV-associated contaminants, such as high abundances of serum proteins, including globulins and albumins, and also non-EV lipid particles such as chylomicrons can influence particle counts and biomarker analysis 155. These lipoproteins of different subpopulations (e.g., very low density, low density, small low density, and high-density lipoprotein) share sizes similar to those of EVs 44. In terms of content, high-density lipoproteins are shown containing miRs, which can disturb the nucleic acid profile of EVs 156. Thus, alternative EV separation approaches such as density gradient centrifugation, SEC, and polymer-based precipitation, with each varying in yield of EVs, the depletion of lipoproteins and protein contaminants, labour-intensity, and cost of the procedure, have been adopted to improve purity ratios and yields of EVs 157. Nevertheless, further study is required to investigate whether EVs from new isolation approaches remain the same therapeutic properties as the traditional one *in vivo*.

## Engineering advanced therapeutic strategies in treating OA and cartilage injury

Injection of specific EVs alone into the articular space may not be optimal towards potential therapeutic outcomes due to low half-lives (<6 hours) *in vivo* 158. Also, the cellular uptake of EVs through several pathways may not be specific to articular chondrocytes. Hence, immune cells can easily uptake the injected EVs, such as dendritic cells (DCs) and macrophages, and other cell types in the microenvironment 159. Although several methods (e.g., 3D culture) have been adopted to enhance the yield of cell-secreted EVs for systematic administration, these limitations are inevitable 160-162. To overcome these barriers, recent research has developed 3D biomaterials/scaffolds for EV retention and to achieve local sustained release. Besides, EVs can be engineered to enhance cell-targeting delivery through employing surface modifications or coupling with nanomaterials. Alternatively, donor cells, such as MSCs, can be pre-conditioned to improve the regenerative potential of the donor cells-derived EVs. This section highlights the advanced strategies that combine molecular biology, biomaterials, and/or nanotechnology to optimize the OA therapy/cartilage repair capacity.

### Engineered EVs as therapeutic agents for joint arthritis

**Figure 3:**
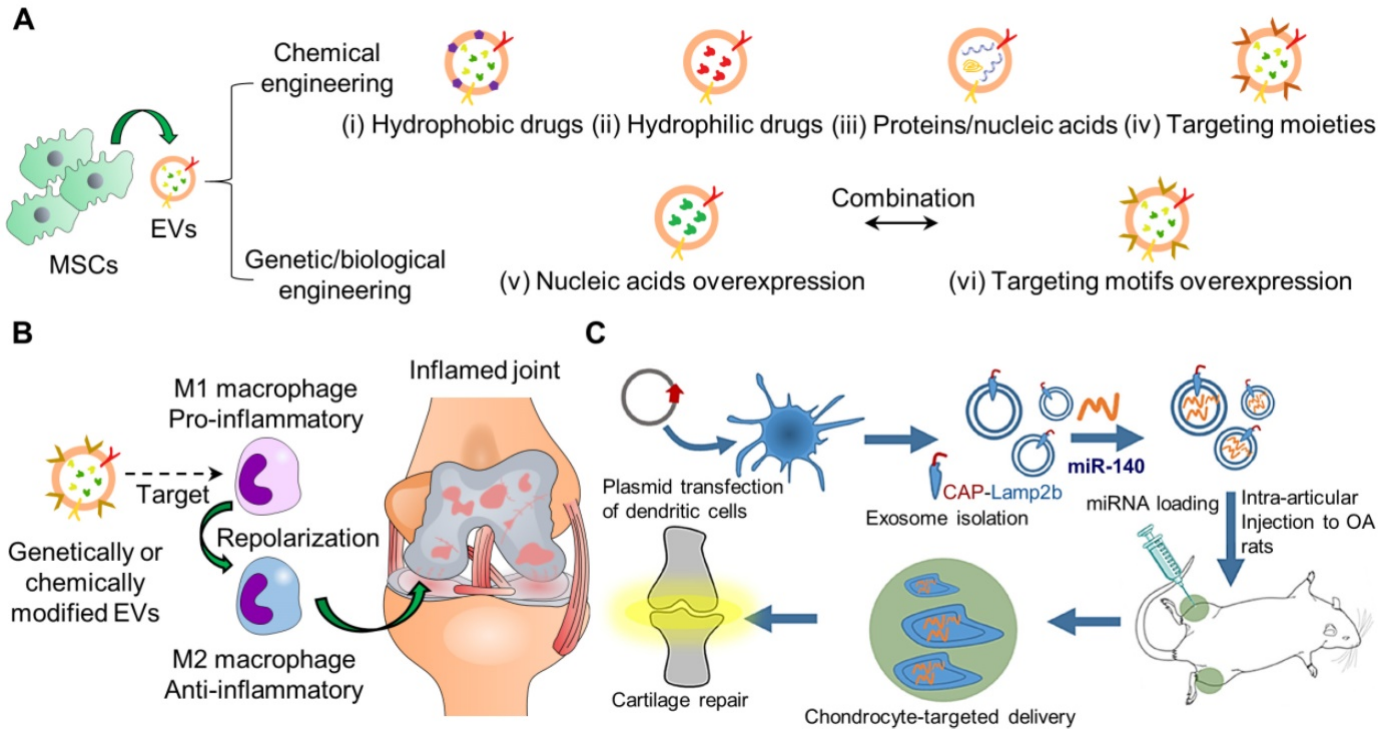
**Schematic representation of engineering EVs for cargo delivery by various types of strategies for OA.** (A) Chemical engineering strategies for the incorporation of (i) hydrophobic drugs, (ii) hydrophilic drugs, (iii) proteins/nucleic acids, and (iv) targeting moieties into EVs. Genetic/biological engineering strategies for (v) nucleic acids and (vi) targeting motifs overexpression in EVs. Multiple strategies may be combined to optimize therapeutic efficacy. (B) Genetically/chemically engineered MSCs-derived EVs can be used for targeting and repolarizing activated macrophages from M1 (pro-inflammatory) to M2 (anti-inflammatory) to treat OA as the recent novel therapeutic approach. (C) Genetically engineering synovial fluid MSCs-derived EVs to target chondrocytes for OA 184. The figures are reprinted with permission from Ref. 184. Copyright American chemical society (2020).

Recent research has attempted to incorporate therapeutic agents into EVs or EVs mimetics through passive or active loading. Passive encapsulation only requires a simple incubation of EVs with desired drugs or cells that can secrete desired drugs spontaneously through hydrophobic interactions 169. However, this approach is low efficiency for loading drugs. Alternatively, active loading involves physical, chemical, and genetic/biological engineering to modify/insert EV contents or surface proteins of EVs (**Figure 3A**). Subsequently, the engineered EVs can carry an elevated level of specific contents or cell-targeting ligands to enhance the therapeutic effects and also insert imaging molecules for long-term tracking purposes. Several reviews have comprehensively described the details of these engineering strategies for targeted drug delivery 42, 170. Nevertheless, many studies have overwhelmingly paid efforts to modify EVs for tumour therapy, probably because of the urgent need to tackle the fast-growing and high lethal rate of cancer in patients 171. The engineered EVs for tumour therapy were also conjugated with targeting ligands and contained a high content of bioactive molecules such as miR, mRNA, and proteins that were shown to suppress tumour cell growth or trigger T-cell response to attack specific tumour cells 172. Also, chemical drugs were loaded into EVs for chemotherapy 173. We expect that more studies will focus on the emerging role of EVs in repairing joint-related diseases in the near future. In this session, we mainly explore a few examples of modified EVs for RA/OA.

As the infiltration of inflammatory cells plays a vital role in cartilage destruction and bone erosion, the modulation of the immuno-environment may help reverse OA progression 174. Conventional methods adopt suppressing the inflammation process, including inhibiting inflammatory cytokines and depletion of M1 macrophages 175. Several reports have employed nanomaterials to re-polarize macrophages from M1 to M2 176-179, but limited studies of this field focus on OA treatment (**Figure 3B**). Recent approaches realize the conversion of dominant phenotype from M1 to M2 to foster a restorative environment in RA 174. He et al. reported the promotion of M2 macrophage polarization by jaw bone marrow MSC (jBMMSCs)-derived EVs (jBMMSC-EVs) through the EV content, miR-223 targeting pknox1. Likewise, Cui et al. also showed that jBMMSC-EVs reduced expression levels of the pro-inflammatory cytokines, IL-1β, IL-6, and TNF-α but increased IL-10 in synovial fluid for M2 macrophage generation 180. To improve the biodistribution half-lives of EVs for the immunomodulation approach, Park and colleagues developed metabolically engineered adipose-derived stem cell EVs (ADMSC-EVs) to target activated macrophages 181. The authors conjugated dextran sulfate to ADMSC-EVs by bio-orthogonal copper-free click chemistry to target the scavenger receptor class A (SR-A) of activated macrophages. The EVs were isolated at a high yield using tangential flow filtration. More importantly, the intravenously injected ADMSC-EVs effectively accumulated in the inflamed joints of mice with collagen-induced arthritis and at higher levels than bare EVs, thereby reducing the administrative dose 10 times for reprogramming of macrophages from M1 to M2. Thus far, engineering EVs for targeting and modifying the dominant phenotype of immune cells is promising for OA therapeutics.

Activated macrophages also express folic acid (FA) receptors (FRs), especially FRβ, on the membrane surface. Thus, FRβ can be a target to mediate cellular uptake 182. Yan et al. reported a platform of RAW 264.7-derived EVs coupled with FA-bearing polyethylene glycol (PEG)-cholesterol (Chol) to encapsulate and control the delivery of dexamethasone (Dex), which is the most frequently used glucocorticoids to treat RA in the clinic. Dex can downregulate pro-inflammatory cytokines of macrophages 183. The incorporation of Dex into EVs was achieved by electroporation. The modified EVs showed prolonged circulation and enhanced RA therapeutic efficacy compared to synthetic liposomes counterparts.

Similarly, EVs can be engineered to target chondrocytes. Xia and colleagues recently demonstrated a chondrocyte-targeted miR-140 delivery platform based on EVs (**Figure 3C**) 184. The authors transfected dendritic cells with plasmids to overexpress chondrocyte-affinity peptide (CAP) with lysosome-associated membrane glycoprotein 2b (Lamp2b), utilized for EV isolation. The authors incorporated miR-140 into the isolated EVs by electroporation. Chondrocytes effectively endocytosed the EVs with increased intracellular miR-140 levels and decreased expression levels of MMP-14 and ADAMTS-5, the markers inhibiting the metabolic balance of chondrocyte matrix *in vitro*. Moreover, the intra-articularly injected EVs remained around the cartilage tissues within the DMM model ~1-fold more than the non-tagged EVs within 24 or 48 h. These findings demonstrate the possibility of genetically modified EV surfaces for prolonged retention in wound sites 185.

CRISPR/Cas9 system has been a promising and powerful gene therapy tool by genome editing 186. It opens new avenues and possibilities for treating OA. Numerous literature reports the nullification or upregulation of different molecular targets of the host MSCs, chondrocytes, or the neighbouring osteoblasts by the CRISPR/Cas9 system to alleviate OA severity 187-194. To tackle with inflammation microenvironment inhibiting cartilage formation, Brinchmann and colleagues knocked out the IL-1β receptor (IL1R1) of human articular chondrocytes and rendered the cells not amenable to secret inflammatory cytokines under IL-1β stimulation 195. Thus, the gene-edited cells can be re-injected into the OA site for improved therapeutic effects. To enable *in vivo* gene editing, effective delivery of the CRISPR/Cas9 system to the target cells is necessary, especially utilizing EVs. However, incorporating large nucleic acids (e.g., CRISPR/Cas9 vectors) into EVs/exosomes is challenging. Lin et al. have developed exosome-liposome hybrid nanoparticles through merging CRISPR/Cas9 expression vectors-bearing liposomes with exosomes at 37 ^o^C overnight to achieve membrane fusion 196. Their results demonstrated that mBMMSCs successfully endocytosed the hybrid nanoparticles to express the CRISPR/Cas9 system for knocking out Runx2 expression. Potentially, the surface of the hybrid nanoparticles can be modified with receptor ligands for targeting specific cell types, such as articular chondrocytes and macrophages *in vivo*.

Besides the mentioned EV modification methods, MSCs can also be primed/pre-conditioned to produce EVs with desirable miR profile/contents useful for OA treatment 197. For instance, curcumin is a natural polyphenol compound derived from turmeric and shows anti-osteoarthritic and anti-inflammatory effects 198. However, several limitations such as low stability, hydrophobicity, and fast systemic elimination restrict its bioavailability. Li et al. have reported a strategy that curcumin-treated hBMMSCs can secret curcumin-containing EVs (Cur-EVs), which can be further harvested for the treatment 199. Their results demonstrated that Cur-EVs were able to upregulate the expression of has-miR-126-3p in IL-1β-stimulated primary human articular chondrocytes with promoted viability, reduced apoptosis, and reduced phosphorylation of components of pro-inflammatory signalling pathways. The authors concluded that upregulated has-miR-126-3p suppressed the pro-inflammatory signallings by MAPK, NF-κB, and PI3K/Akt, which controlled the pathways participating in the progression of OA. These findings prove the anabolic effects of Cur-EVs derived from curcumin-treated hBMMSCs on OA. Similarly, Rong and colleagues reported that hypoxia (hypoxia-inducible factor-1α)-treated BMMSCs released EVs with a high expression level of miR-216a-5p to promote anabolism, migration, proliferation, and apoptosis inhibition of IL-1β-induced rat joints-derived chondrocytes *via* suppressing JAK2/STAT3 pathway, which was shown to play a pathological role in OA 200. Their findings also showed that intra-articular injection of the hypoxic hBMMSCs-derived EVs effectively attenuated the cartilage degeneration in the DMM-triggered OA model. Therefore, rather than performing sophisticated engineering methods, the applications of EVs derived from those preconditioned/primed MSCs may provide a simple route to obtain a promising drug delivery vehicle of useful contents for the treatment of OA.

### Biomaterials for EV retention and delivery

The therapeutic efficacy of EVs is directly related to the exposure time of EVs to targeted cells and surface receptors. Similar to the challenges with cell or soluble factor delivery, EV delivered intravenously, intraperitoneally, or *via* subcutaneous injection are rapidly cleared *in situ* by circulating innate immune cells and subsequently require repeated administration to obtain their desired effect 201. Biomaterials have made a significant impact on facilitating the local and sustained delivery of therapeutic agents over the last 60 years (extensively reviewed in 202), and have become a promising and attractive approach for mitigating the clinical barriers of EV translation *in vivo* 203.

Extracellular matrix-derived natural biopolymers and proteins found in native tissues such as hyaluronic acid, sulfated glycosaminoglycans, collagen, and fibronectin contain motifs have been exploited to retain EVs *via* binding or affinity-based mechanisms locally 204. Previous work has shown that MSC-EVs bind to collagen and fibronectin *via* integrins and to hyaluronic acid *via* CD44 interacting with hyaluronan 73, 205. While natural polymers have unique biomimicry and bioactivity, one of the main disadvantages to their use is that raw materials may have significant innate variability depending on tissue source/origin 206. On the other hand, synthetic polymers tend to have less raw material variability related to supply availability or batch-to-batch consistency, are associated with lower costs, but lack inherent composition/structure to interact with cells as natural polymers do 207. Synthetic and naturally-derived polymers can be chemically modified to facilitate material tunability with respect to architecture (shape, size, pore/mesh size, topography), degradability, bioactivity, biocompatibility, and mechanical behavior 208-210. Each of these parameters plays a significant role in the therapeutic administration of the biomaterial (i.e., implant vs. injectable), is specific to the targeted tissue/injury site, and will subsequently impact EV delivery.

**Figure 4:**
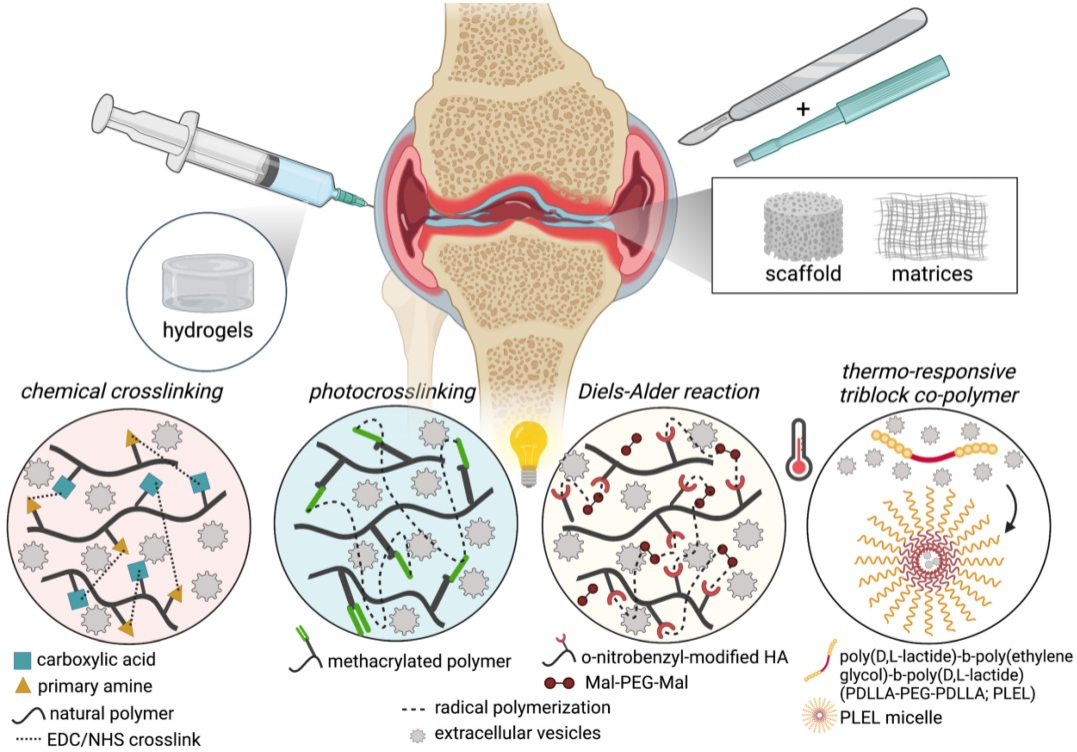
**Summary of EV-biomaterials for cartilage tissue engineering or treatment of OA**. Biomaterials for OA include implanted biomaterials and intra-articularly injected biomaterials into joints induced with OA (e.g., DMM or osteochondral defects). Biomaterials are generated from natural polymers/materials such as decellularized cartilage tissue, hyaluronic acid, gelatin, collagen, chitosan, nanoclay, or even synthetic polymers, including PEG. Studies feature various strategies to modulate biomaterial tunability and EV retention and releases, such as chemical crosslinking, chemical modification of natural polymers to enable photocrosslinking or thermoreversibility, and even synthetic thermoresponsive triblock copolymer that forms micelles at room temperature.

Biomaterials of various forms, including hydrogels, sponges, membranes/matrices, scaffolds, and decellularized tissues, have been used to incorporate and deliver EVs. Most commonly used biomaterials for EV release are hydrogels and ECM-based scaffolds/matrices; hydrogels are (synthetic or natural) polymer networks swollen in water with very well-established and diverse fabrication techniques, chemical modifications, drug delivery release/diffusion kinetics, while ECM-based materials (e.g., decellularized tissues, matrix fibers) retain native architectures and chemical compositions and also have a variety of well-established fabrication methods to produce complex 3D structures and drug-loading. Natural or synthetic polymers can be combined to generate hybrid or composite biomaterials with more tunable functionality with respect to fabrication, physical or mechanical properties, or the addition of therapeutics. Chemical modifications of biopolymers for hydrogel formulation have been extensively reviewed 211. Biopolymer modifications used for EV retention and delivery for cartilage tissue engineering applications, including the incorporation of biopolymers and carbodiimide crosslinking 212, 213, photocrosslinkable methacrylamide-modified gelatin (GelMA) 214, 215, modifications to support thiol-ene reactive Michael addition crosslinking 216, dynamic covalent crosslinking via reversible Diels-Alder reactions 217, and synthetic thermosensitive triblock copolymers (**Figure 4**) 218. The use of various kinds of biomaterials for EV delivery for tissue regeneration in several applications has been thoroughly reviewed 203, 219-224. In this section, we will provide a brief overview of ECM-based scaffolds and hydrogels that have been used for EV delivery, including the rationale for their use, along with a description of form and function with respect to therapeutic delivery in this session.

#### EVs-biomaterials for cartilage tissue engineering or treatment of OA

While there are numerous studies (and reviews) of EV delivery *via* biomaterials for bone regeneration 225-227, cardiac tissue remodeling following myocardial infarction 228, and traumatic brain injury 229, there are only a small number of studies that have specifically investigated EV delivery from biomaterials for cartilage tissue regeneration or treatment of osteoarthritis. A few major themes emerge when examining these studies collectively. Biomaterials are primarily fabricated in scaffold or hydrogel form, are derived from natural polymers such hyaluronic acid or gelatin (one exception where a synthetic triblock copolymer hydrogel was investigated), and are applied within animal models surgically as an implant or *via* intra-articular injection. Additionally, a full-thickness osteochondral defect in New Zealand rabbits is the most heavily used animal model to assess treatment efficacy for cartilage regeneration, followed by an osteoarthritis model transecting the anterior cruciate in combination with a medial meniscectomy in Sprague-Dawley rats. Lastly, all studies EVs derived from human cells (primary or cell line) plated on tissue culture plastic used ultracentrifugation for isolation, collection, and a combination of nanoparticle tracking analysis or dynamic light scattering, transmission electron microscopy (TEM), and western blotting or flow cytometry for EV characterization prior to use *in vivo*. Experimental details, including EV source, isolation technique, biomaterials, *in vivo* models, and outcomes, are summarized in **Table 3**.

#### Implantable scaffolds for EV delivery

Decellularized extracellular matrix continues to be a widely used and attractive raw material for biomaterial scaffold fabrication due to the retention of native proteins and matrix architecture while effectively eliminating cells or debris known to cause a detrimental immunological response *in vivo*. While there are challenges in working with decellularized materials as described above, combining decellularized tissues with synthetic or chemically modified natural polymers has enabled a high level of tunability, bioactivity, and manufacturability. For example, decellularized porcine cartilage combined with GelMA was used to generate a unique 3D printable bioink to support the controlled release of MSC-EVs 214. Prior to evaluating a novel 3D printed material, Chen et al. investigated the efficacy of EVs in modulating chondrocyte behavior and found that isolated EVs alone promoted decreased expression of MMP-13 (a marker of cartilage degradation) and ADAMTS-5 while also increasing COL2A1 and aggrecan expression 214. Proteomic analysis followed by subsequent gene ontology enrichment analysis and STRING showed that EVs were significantly enriched in various segments of the mitochondria, suggesting that these processes and pathways may be heavily involved in the chondroprotective function of the EVs. Using an inhibitor to induce mitochondrial damage to chondrocytes *in vitro*, EVs were found to provide unique mitochondrial proteins to rescue the damage 214. Next, dye-labeled EVs were incorporated into the decellularized cartilage ECM-GelMA bioink and 3D printed using a desktop stereolithography technique in combination with visible light crosslinking initiated by LAP to fabricate high-resolution hydrogel scaffold discs with radially oriented channels and a pore size between 100-500 µm to facilitate optimal cell infiltration. A pilot subcutaneous investigation showed that EVs were retained within the 3D printed scaffold for up to 14 days promoted significantly fewer M1 macrophages (CD86 and CD3) and increased M2 macrophages (CD163 and Arg1) compared to scaffolds without EVs. Subsequent *in vivo* analysis in a rabbit osteochondral defect model showed that the EV-containing hydrogel significantly increased ICRS macroscopic scores (Safranin O and alcian blue) and immunohistochemistry of COL2A1 and decreased MMP13 expression after the 6 and 12 week time points compared to all other groups, demonstrating a clear impact of local EV retention for cartilage tissue regeneration.

Surface modification of biomaterial scaffolds with EVs or employing a biomaterial scaffold as a reloadable depot for EV retention and release are other methods recently investigated for cartilage tissue engineering. EVs isolated from human umbilical Wharton's jelly MSCs (gelatinous substance on the inside of the cord that is rich in hyaluronic acid and chondroitin sulfate) intra-articularly injected weekly into rabbit osteochondral defects pre-implanted with freeze-dried, and EDC-crosslinked decellularized porcine cartilage scaffolds enhanced osteochondral regeneration compared to the scaffold alone 212. A follow-up analysis was subsequently performed to investigate potential mechanisms of action of EVs compared to saline control in the osteochondral defect model and found that EVs did not significantly change the pro-inflammatory expression of TNF-α or IL-1β. However, the EVs show significantly increased IL-10 staining in the defect and synovium after 10 days, suggesting EVs promoted increased anti-inflammatory behavior 212. Furthermore, EV treatment promoted significantly higher numbers of M2 macrophages (CD206+), lower numbers of M1 macrophages (CD86+), with no significant changes in MSC proliferation or endogenous recruitment after 10 and 20 days post-treatment.

#### Injectable hydrogels for EV delivery

HA hydrogels have recently been investigated in conjunction with EVs for enhanced cartilage repair and regeneration, including soluble high molecular weight HA viscosupplements alone. Treatment of critical-sized osteochondral defects in five-month-old New Zealand White rabbits immediately following wound closure with either 1mL intra-articular injections of high molecular weight HA (1100 kDa, 3 wt%) or 1mL of high molecular weight HA and 200 µg of EVs isolated from clonal immortalized E1-MYC 16.3 human embryonic stem cell-derived MSC line, with repeated injections performed 7 and 14 days post wound closure resulted in significantly increased ICRS score at 6 and 12 weeks 230. Further, combined HA and EV treatment resulted in significantly increased toluidine blue (sulfated glycosaminoglycans) and decreased collagen I staining after 12 weeks (but no significant differences at 6 weeks) compared to HA alone, which also yielded more fibrocartilage rather than hyaline cartilage 230. This work demonstrates that repeated intra-articular injection of high molecular weight HA combined with EVs improves cartilage tissue repair and regeneration compared to HA alone; however, it is unclear how long EVs are retained within the material or the dose at which they are released. Other studies have investigated the use of covalently crosslinked HA, modified HA and/or supplementing HA with other biopolymers to augment material functionalization and incorporate EV delivery.

##### Chemical crosslinking

Freeze-dried EDC/NHS-crosslinked chitosan and hyaluronic acid hydrogels (CS-HA) combined with EVs and adipose-derived MSCs injected into full-thickness osteochondral defects immediately following surgery promoted significantly increased ICRS scores of MRI imaging compared to the hydrogel alone, MSCs alone, MSC-EVs, CS-HA/MSC, or the CS-HA/MSC-EVs group after 4 weeks and 24 weeks 213. Further, gross analysis and scanning electron microscopy of the joint surface, along with histological analysis (hematoxylin and eosin, Masson's trichrome, and ColII) after 24 weeks illustrated the CS-HA/MSC and CS-HA/MSC-EVs group produced cartilage repair with ICRS scores that were not statistically different from normal cartilage 213.

##### Photocrosslinkable hydrogels

HA modified with o-nitrobenzyl alcohol (HA-NB) generates aldehyde groups under light irradiation that can interact with amines on other biopolymers, enables *in situ* covalent crosslinking; this design was recently exploited by Liu et al. to entrap and deliver EVs derived from human induced pluripotent (cell line iPS-S-01)-derived MSCs to cartilage defects in rabbits 216. Full-thickness cartilage defects filled with HA-NB, combined with gelatin and EVs (EHG), photo-irradiated for one minute at 395 nm integrated within the native cartilage matrix, promoted cell deposition, and cartilage defect repair significantly better than the HA-NB/gelatin (HG) hydrogel alone, pre-cultured EHG implants, or intra-articular injections of EVs at the same dose alone. Importantly, this study examined EV retention by immersing 200 µL gels prepared with 2.45^12^ DiI-labeled EVs in fresh PBS daily; supernatants were analyzed with a particle analyzer and subtracted from the loaded EV total to determine percent retention 216. There are limitations to this technique, as it is unclear if the temperature or mechanical agitation were used during the EV release study or if the physical properties of the hydrogel glue (e.g., degradation, swelling) influenced EV retention/release. In another study, authors used a BCA protein assay to quantify the release of small EVs isolated from human umbilical cord MSCs from photo-crosslinked (3 minutes, UV light) GelMA/laponite nanoclay hydrogels (nanoclay is a unique nanoparticle composite material containing layered silicates) 231; the GelMA/nanoclay photocrosslinked hydrogel retained EVs for up to 30 days *in vitro* compared to crosslinked GelMA alone or GelMA/gelatin hydrogels 215. Furthermore, the EV-containing hydrogel significantly improved cartilage repair in a full-thickness osteochondral defect in Sprague-Dawley rats compared to the Gel-nano hydrogels alone after 12 weeks, suggesting the retention and subsequent release of EVs over 30 days provided the enhanced therapeutic effect 215. Hu et al. also performed extensively *in vitro* mechanistic analysis on their EVs; microarray analysis demonstrated that miR-23a-3p was highly enriched, and bioinformatic analysis suggested that miR-23a-3p may bind to the 3'UTR coding sequence of the gene PTEN. The authors then cultured miR-23a-3p with 293 T-cells transfected with luciferase reporter constructs containing the predicted 3'UTR of PTEN and found that luciferase activity was decreased and subsequently abolished when cultured with mutated 3'UTR of PTEN, confirming EV function via miR-23a-3p targeting PTEN 215. A silencing assay with human bone marrow-derived mesenchymal stem cells further validated that miR-23a-3p attenuated the effects of EVs on COL2A1 and SOX9 gene expression, alcian blue staining, and impaired activation of protein kinase B (AKT) secretion *via* western blotting. Collectively, these two studies highlight the clinical efficacy of using photocrosslinkable networks for EV retention and delivery from hydrogels.

##### Thermo-responsive hydrogels

As previously described, covalent/supramolecular interactions can be exploited to entrap and control the delivery of biomolecules such as EVs. Here, Diels-Alder crosslinked hyaluronic acid/PEG hydrogels (DAHP) were developed using a furyl functionalized HA and a maleimide (Mal)-PEG-Mal crosslinker that enables orthogonal crosslinking with low reactivity with amine groups 232. Diels-Alder crosslinked hydrogels are thermo-reversible polymer networks formed without any additional catalyst of toxic solvent, making them particularly advantageous for incorporating biomolecules 233. Wang et al. found that DiO-labeled EVs isolated from human-induced MSCs derived from iPSC (C1P33) were released from the DAHP hydrogel for 16 days *in vitro* via a transwell assay (0.4 µm membrane, ~16% cumulative release) and nanoflow cytometric analysis. Furthermore, EV release kinetics were accelerated under increasing concentrations of hyaluronidase treatment, confirming a degradation-dependent control of EV release 232. Using a model of OA in rats (transection of the anterior cruciate ligament in combination with partial medial meniscectomy), authors found that intra-articular injection of hydrogels containing EVs significantly improved cartilage repair (the lowest OARSI score) compared to the DAHP hydrogel alone and a single injection of EVs after 35 days *in vivo* 232. Interestingly, the authors also found minimal differences in cartilage repair between the hydrogel delivery and weekly intra-articular injections of EVs, suggesting the good potential of hydrogels as a single-application therapeutic.

In addition to EVs being a potent and independent therapeutic for treating OA or promoting cartilage repair, EVs have also been shown to be unique bioactive carriers for nucleic acids 234. Tao et al. showed that sleep-related circular RNAs (circRNA), which are more stable and less susceptible to rapid clearance than miR or linear RNAs 235, are enriched in melatonin-treated chondrocytes that are thought to play a role in OA pathogenesis 218. The authors isolated EVs from synovium mesenchymal stem cells (SMSCs) overexpressing circRNA3503 and loaded them into a poly(D, L-lactide)-b-poly(ethylene glycol)-b-poly(D, L-lactide) (PDLLA-PEG-PDLLA; PLEL) triblock copolymer hydrogels. This thermosensitive triblock copolymer is particularly useful as an injectable vehicle for biotherapeutics due to its ability to self-assemble into micelles at room temperature and non-flowing structure under physiological load 218. Using a model of OA in rats (transection of the medial collateral ligament, medial meniscus, and anterior cruciate ligament), the PLEL hydrogels loaded with circRNA-doped EVs demonstrated significantly enhanced cartilage repair after 24 weeks (injections performed after surgery and every 4 weeks after OA-induced injury) compared to the PLEL hydrogel alone or PLEL hydrogel with SMSC-EVs *via* Safranin O/Fast Green and Toluidine Blue, and Col II staining 218. EVs released from hydrogels were confirmed *in vitro* over 36 days performed on a rocker at 37 ºC and detected using a CD63 ELISA kit. Mechanistic *in vitro* studies of the circRNA3503-overexpressed EVs highlighted their efficacy in attenuating inflammation-induced apoptosis and provided a balance between ECM synthesis and degradation by acting as a sponge for miRs that regulate expression of target functional genes such as MMPs and SOX9 218.

### Summary of biomaterials for EV/exosome delivery

Together, these studies showcase a wide variety of biomaterials for EV delivery to promote cartilage tissue repair and regeneration. Collectively and independently, each of these materials indicates potential for their biomaterial delivery vehicle to release EVs and subsequently enhance cartilage repair compared to the materials alone and in some cases compared to EV treatment alone. As discussed in many review articles, there are significant limitations to translating EV/biomaterial therapeutics to clinical application, first, in standardizing cell/tissue/species source for EV generation, scalability, isolation techniques, application techniques (e.g., single therapeutic application or application of an EV/biomaterial therapy followed by multiple injections of EVs at later time points) and in designing appropriate pre-clinical studies to thoroughly identify and qualify that the effects of EV-loaded materials are primarily driven by EVs. Second, there also remains challenges in standardizing techniques to evaluate EV release and subsequent synergistic effects of bioactivity of EVs released from biomaterials. Important to note that many of the specific functional outputs *in vitro* and *in vivo* be tissue- or application-specific (i.e., cartilage vs. cardiac vs. bone). Decoupling these effects will be critical to validate EV/biomaterial efficacy.

Lastly, based on several reviews and publications showing the robust effects of intra-articular delivery of EVs to promote cartilage repair and regeneration, studies here suggest that a single application of EV-loaded biomaterials may provide a similar therapeutic effect controlled *via* material degradation-mediated EV release or EV diffusion from biomaterials. It will be essential to elucidate the effects of EV/biomaterial degradation further and release with respect to timing and dosing to achieve a therapeutic effect for OA. One area of biomaterials that has shown promise in cartilage tissue engineering (and many other applications) that may be advantageous for EV delivery is granular hydrogels and microparticles 236, 237. These materials are uniquely poised for various biopolymer compositions, fabrication methods, and tuning therapeutic delivery that can be applied in the joint using translational and minimally invasive surgical techniques.

## Conclusion and Perspective

In summary, recent research has made significant progress in overcoming major barriers to using EVs as a delivery system and a marker for OA pathology diagnosis. EVs are ideal systems for delivering osteoarthritis therapeutics, owing to their size, surface expression profiles, low immunogenicity, low cytotoxicity, and long-term safety. EVs from modified cells or engineered EVs with drug loading technologies have been shown to improve the therapeutic effect. However, the side-effect of using EVs for OA therapy is unknown, and safety evaluation research is required before clinical translation. Recent advances in nanomaterials-based offer great sensitivity and rapid biosensors for detecting EVs. Tissue engineering techniques are also used in EV-based OA therapy/cartilage repair. Biological scaffolds, especially hydrogels, have been shown to have a good sustained EV-release effect in cartilage repair. We believe that theranostic platforms will be the main direction of EV-based OA therapy/cartilage repair in the near future.

The mentioned exosome-liposome nanohybrid system also inspires us to consider its potential application to detect both EVs and the content of EVs 196. For instance, the liposome surface can be modified with EV binding peptide/aptamers that will turn on the signals upon binding with EVs. In parallel, biosensors for sensing EV contents, such as miR, can be initially encapsulated in the liposomes, and another physical/chemical signal will be switched on upon the liposomes fusing with EVs/exosomes. Thus far, this nanosystem can simultaneously probe both EVs and OA biomarkers in EVs. Nevertheless, this platform will require further research to validate efficacy.

## Figures and Tables

**Table 1:** Biomarkers in EV-derived from the serum/SF of patients with joint arthritis.

EV-derived biomarkers	EV Source	Expression levels	Possible biological effects/reasons	Source of OA joints	Reference
*microRNAs (miR)*					
miR-126-3p	Human synovial fluid	Downregulation in OA patients	*In vivo*, rat SFC-derived EVs containing miR-126-3p could constrain chondrocyte inflammation and cartilage degeneration	SF from knees of OA patients undergoing TKR	73
miR-500b miR-720 miR-4454 miR-199b-5p miR-3154	Human synovial fibroblasts	Upregulation with IL-1β stimulation	All of them presented in IL-1β-stimulated SFB and EVs from IL-1β-stimulated SFB	Normal human knee synovial fibroblasts and chondrocytes	111
miR-504-3p miR-16-2-3p miR-210-5p miR-26a-5p miR-146a-5p miR-6821-5p miR-68678-3p	Human synovial fluid	Upregulation Upregulation Upregulation Downregulation Downregulation Downregulation Downregulation	miR-504-3p is the only common miR upregulated in both male and female OA patients, highly gender-specific.	Normal/OA SF was obtained from knee joints of patients undergoing arthrocentesis/ TKR	112
miR-372-3p	Human chondrocytes	Upregulation in OA chondrocytes	Promoted cell growth and proliferation GSK signalling pathway	Human cartilage specimens were obtained from patients undergoing TKR	66
miR-449a-5p	Human primary chondrocytes	Upregulation with IL-1β treatment	Inhibit ATG4B expression and autophagy in LPS-primed macrophages	Human cartilage specimens were obtained from patients undergoing TKR	67
miR-155-5p	Human synovial fluid	Upregulation	Potentially stimulate a positive feedback loop of TNF-α stimulated inflammation	SF was obtained from knee joints of patients (ages of 40-60) undergoing arthrocentesis	113
*Long non-coding RNA (lncRNA)*					
PVT1	Human serum	Upregulation	EV-derived PVT1 regulated OA progression by modulating the HMGB1/Tlr4/NF-κB pathway	Whole blood was extracted from 30 OA patients (ages range from 50-70 years old) and 30 healthy volunteers (ages range from 50-70 years old)	74
HULC	Human chondrocytes	Downregulation in OA chondrocytes	Suppressed cell growth and proliferation GSK signalling pathway	Human cartilage specimens were obtained from patients undergoing TKR	66
PCGEM1	Human synovial fluid	Late-stage OA > early-stage OA > Control	Distinguish the stage of OA. There was a positive relationship between EV-derived lncRNA PCGEM1 and WOMAC Index	Blood sample from the cubital vein and synovial fluid sample from knee joints: (1) 20 healthy people who suffered from incidental knee pain as a control group; (2) 20 patients with primary OA in the early stage; (3) 22 patients with primary OA in the progressive stage (late-stage)	70
*Proteins*					
Haemoglobin Actin-related protein 2/3 complex subunit 3	Human synovial fluid	Upregulation	More abundant in OA than those in RA, spondyloarthritis (axSpA), and gout	SF-derived EVs were isolated from RA, axSpA, gout, and OA patients	75
COL6A1 Β-2glycoprotein I Complement component 5-variant Haptoglobin Alpha-1-acid glycoprotein Ceruloplasmin KIAA1466 CCDC101 PPARBP Apolipoprotein Anti-folate binding protein Anti-HER3 HRV Fab N27-VL C1QC	Human synovial fluid	Upregulation (Male) Upregulation (Male) Upregulation (Male) Upregulation (Female) Upregulation (Female) Upregulation (Female) Downregulation (Male) Downregulation (Male) Downregulation (Male) Downregulation (Female) Downregulation (Female) Downregulation (Female) Downregulation (Female) Downregulation (Female)	The upregulated or downregulated markers were gender-dependent in EV protein cargo from SF of non-OA and OA patients	Knee joint synovial fluid from both healthy and osteoarthritic knees was obtained from patients (8 non-OA females, 10 OA females, 7 non-OA male, and 7 OA male patients) undergoing arthrocentesis/total knee arthroplasty procedures.	114
Toll-like receptor 3 (TLR3)	Human serum	24- and the 17- to 18-kDA TLR3 showed ~6-fold higher intensity in the active RA group than in the other groups	The increased TLR3 expression in active RA patients might reflect the inflammatory conditions of fibroblast-like synoviocyte	Whole blood was extracted from 33 patients (12 with active RA, 11 with inactive RA, 10 with OA, and 10 healthy donors)	115

**Table 2:** *In vivo* efficacy of MSC-EVs in animal cartilage/osteochondral models.

EV source	Dose/volume	Animal type	Disease model	Molecular mechanisms	Biological Outcomes	Reference
IPFP-MSCs*	10 μl 10^10^ particles mL^-1^	Mice	Surgical destabilization of the medial meniscus (DMM)	Inhibition of mTOR	Prevent the cartilage destruction and partially improve the gait abnormality in the DMM mice model	134
human embryonic stem cell-derived MSCs	100 µg EVs per 100 µl of injection	Rat	Osteochondral defect model created on thetrochlear grooves of the distal femurs	CD73-mediated adenosine activation of AKT and ERK signallings.	Improve the surface regularity and integration with host cartilages, improve the quality of osteochondral repair	163
hBMMSCs	15 μl 500 μg mL^-1^ in PBS	Mice	Collagenase VII-induced OA model	MiR-92a-3p directly targets the 3'-UTR of WNT5A mRNA	Inhibit the progression of early OA, prevent the damage to knee articular cartilage	164
mBMMSCs	250 ng per 5 µL	Mice	Collagenase VII-induced arthritis model	Re-induce the expression of chondrocyte markers while inhibiting catabolic and inflammatory markers	Higher bone volume (BV/TV parameter); less bone degradation	165
hBMMSCs	100 μL of 10^11^ particles mL^-1^	Mice	Mouse model of traumatic OA in a mechanical test device	miR-136-5p target ELF3, downregulate its expression	Higher expression of collagen II and aggrecan inhibits early post-traumatic OA and prevents further damages to the knee cartilage.	166
mBMMSCs*	200 μg of EVs 200 in μl PBS	Mice	Lumbar facet joint (LFJ) osteoarthritis model	Suppressing RANKL‐RANK‐TRAF6 Signalling Pathway	Attenuate the articular Cartilage degeneration, promote cartilage and subchondral bone remodeling	167
hBMMSCs*	250 ng per 5 µL	Rat	left knee joints of the rats were opened to expose the joints, followed by skin suture	miR-26a-5p specifically target PTGS2	Alleviate synovial tissue proliferation, reduced inflammatory cells, and attenuated pathological changes of synovial tissues	168
Synovial MSCs	100 μL; 10^11^ EV particles mL^-1^	Rat	Transecting the medial collateral ligament and the medial meniscus completely	YAP activation via the alternative Wnt signalling pathway	Slow the progression of early OA and preventedsevere damage to knee articular cartilage	135
UCMSCs	100 μL; 1 mg mL^-1^	Rat	A drill bit (1.5 mm diameter) was used to make cartilage defects on the distal femurs	UCMSC-EVs contain high content of lncRNA H19	Promote chondrocyte proliferation and matrix synthesis and inhibit apoptosis in vitro; promote cartilage repair *in vivo*	148

*represent the EVs are collected from modified MSCs. Abbreviations: murine bone marrow mesenchymal cells (mBMMSCs); infrapatellar fat pad (IPFP) MSCs; human bone MSC (hBMMSC); umbilical cord mesenchymal stem cells (UCMSCs).

**Table 3:** Scaffold/Matrix and hydrogel biomaterials for EV/exosome delivery for cartilage tissue engineering or treatment of OA

EV Source + Isolation Method	Biomaterials	*In vivo* EV dose/volume	*In vivo* model/ Species	*In vivo* timepoints	*In vivo* outcomes	Ref.
**Scaffold/Matrix Biomaterials (Implanted)**						
Bone marrow derived MSCs + tissue culture plastic (TCP) 50-60% confluence + 1hr 100k xg ultracentrifugation (UC)	3D printed decellularized porcine cartilage/GelMA scaffold	200 µg in 200 µL (PBS Control); 200 µg/mL hydrogel	Osteochondral defect in patellar groove; 4mm diameter x 4mm deep; Rabbit	6 and 12 weeks	EV/hydrogel significantly increased ICRS macroscopic scores, COL2A1 expression, and decreased MMP13 expression after the 6 and 12 weeks compared to all controls.	214
Passage (p) 3-5 human umbilical cord Wharton's Jelly MSCs + TCP (60% confluence) + 2hr, 100k xg UC	Freeze-dried decellularized porcine cartilage ECM	25 μg/mL, supplementary EV-only injection once every 7 days for a total of 5 injections	Osteochondral defect in femoral trochlea; 3.5 mm diameter x 1.5 mm deep; Rabbit	12 and 24 weeks	EVs enhanced the effect of the scaffold and promoted osteochondral regeneration; EVs promoted the polarization of macrophages toward the M2 phenotype and inhibited the inflammatory response *in vivo.*	212
**Hydrogel Biomaterials (Injected)**						
Immortalized E1-MYC 16.3 human embryonic stem cell-derived MSCs Size-fractionated, concentrated 50× by tangential flow filtration (100kDa MWCO)	Hyaluronic acid hydrogel solution	200 µg of EVs in 1 mL intra-articular injection days 7 and 14 after wound closure	Osteochondral defect in femoral trochlear grooves; 4.5 mm diameter x 1.5 mm depth; Rabbit	6 and 12 weeks	The combination of MSC-EVs and HA *via* intra-articular injections (immediately post-surgery and after 7 and 14 days) promoted enhanced functional cartilage repair compared with HA alone.	230
Human articular chondrocytes + TCP + 2hr, 100k xg UC	Chitosan-hyaluronic acid hydrogel	30 µg EV + 1.5^6^MSCs + 100 µl hydrogel	Osteochondral defect in patellar groove; 4 mm diameter x 3 mm depth; Rabbit	4 and 24 weeks	EDC/NHS cross-linked CS-HA/EV/MSC, and CS-HA/MSC hydrogel enhanced cartilage repair compared to EV/MSC or CS-HA controls via MRI and histological analysis.	213
P4 human iPSC-MSCs + TCP (80% confluency) + 2hr, 100k xg UC	*o*-nitrobenzyl alcohol-modified hyaluronic acid and gelatin	1^11^ EVs/ml, 20 uL	Osteochondral defect in patellar groove; 4 mm diameter x 3 mm depth; Rabbit	12 weeks	Increased defect regeneration and well-organized articular cartilage structure in the EV/hydrogel group compared to gel alone and EVs alone.	216
Human umbilical cord MSCs + TCP + 70min, 100k xg UC	GelMA and nanoclay composite	1^9^ EVs/mL, volume not specified	Osteochondral defect; 2.5 mm diameter x 1.5 mm depth; Rat	12 weeks	EV delivery increased collagen II stainings compared to controls *in vivo.*	215
p5-10 human iPSC-line C1P33 + TCP + 70min, 100k xg UC	Diels-Alder crosslinked hyaluronic acid/PEG (DAHP) hydrogel	1^10^ EVs/mL (100 uL), supplemental intra-articular treatment; Multi-*treatment* group received injections on 7, 14, 21, 28 days or a single injection at 7 days after surgery	OA model; transection of the anterior cruciate ligament in combination with partial medial meniscectomy; Rat	35 days	DAHP hydrogel improved the bioavailability and therapeutic efficacy of MSC-EVs for OA - with the lowest OARSI score following *in vivo* study.	217
Human synovial membrane stem cells + TCP (50-60% confluency) + 30% sucrose/D_2_O cushion + UC, 1hr 100k xg	Thermoresponsive triblock PDLLA-PEG-PDLLA hydrogel (PLEL)	1^11^ EV/mL (200 µL) + 800 µL of hydrogel solution; Intra-articular injection performed every four weeks after surgery	OA model; transection of the medial collateral ligament, medial meniscus, and anterior cruciate ligament; Rat	24 weeks	PLEL@circRNA3503-OE-sEVs limited OA progression; Through multiple pathways, circRNA3503-OE-EVs alleviated inflammation-induced apoptosis and the imbalance between ECM synthesis and ECM degradation by acting as a sponge of hsa-miR-181c-3p and hsa-let-7b-3p.	218

## Abbreviations

OA: osteoarthritis
MSCs: mesenchymal stem cells
ECM: extracellular matrix
HSP70/90: heat shock proteins 70/90
Col: collagens
Col II: Type II collagen
Col IX: collagen IX
ESCs: embryonic stem cells
EVs: extracellular vesicles
COVID-19: coronavirus disease 2019
miR: microRNAs
mRNAs: messenger RNA
TSG101: tumour susceptibility gene 101
RA: rheumatoid arthritis
HA: hyaluronan
SF: synovial fluid
IncRNA: long non-coding RNA
circRNA: circular non-coding RNA
NTA: nanoparticle tracking analysis
aRT-PCR: quantitative reverse transcription-polymerase chain reaction
RNA-seq: RNA-sequencing
ELIA: enzyme-linked immunosorbent assay
WB: western blotting
SERS: surface-enhanced Raman scattering
SPR: surface plasmon resonance
ICA: immunochromatographic assay
CL: chemiluminescence
ECL: electrochemiluminescence
PRP: platelet-rich plasma
PDGF-AB: platelet-derived growth factor-AB
TGF-β: transforming growth factor-β
VEGF: vascular endothelial growth
IL-1β: interleukin-1β
IPFP: infrapatellar fat pad
AC: articular chondrocytes
NP: nanoparticles
HLA-DR: human leukocyte antigen-DR
IFN-γ: interferon gamma
MMP-13: matrix metallopeptidase 13
ADAMTS5: a disintegrin and metalloproteinase with thrombospondin motifs
SMSCs: synovial MSCs
H3K4: histone H3 at lysine 4
KDM2A: lysine demethylase 2A
SFRP2: secreted frizzled-related protein 2
E2F1: E2F transcription factor 1
TNF-α: tumour necrosis factor-α
RalA: ras-related protein
BMMSCs: bone marrow MSC
ADMSC-EVs: adipose-derived stem cell EVs
FA: folic acid
PEG: polyethylene glycol
Chol: cholesterol
Dex: dexamethasone
CAP: chondrocyte-affinity peptide
Lamp2b: lysosome-associated membrane glycoprotein 2b
HA: hyaluronic acid
RHAMM: hyaluronan-mediated motility receptor
GelMA: methacrylamide-modified gelatin
EDC: 1-ethyl-3-(3-dimethylaminopropyl)carbodiimide-HCl
NHS): n-hydroxysuccinimide
TEM: transmission electron microscopy
Arg-1: arginase-1
AKT: activation of protein kinase B (AKT)
OARI: osteoarthritis research society international
PLEL: poly(D, L-lactide)-b-poly(ethylene glycol)-b-poly(D, L-lactide)
OARSI: Osteoarthritis Research Society International
